# Low-calorie diet intervention ameliorates gut microbiota dysbiosis and metabolic changes in obese patients with type 2 diabetes under standard care

**DOI:** 10.1016/j.csbj.2025.11.043

**Published:** 2025-11-20

**Authors:** Mongkontida Umphonsathien, Pornsawan Prutanopajai, Thanya Cheibchalard, Naraporn Somboonna

**Affiliations:** a Division of Endocrinology and Metabolism, Department of Medicine Police General Hospital, Bangkok 10330, Thailand; b Department of Executive and Research Office, BDMS Wellness Clinic, Bangkok 10330, Thailand; c Department of Microbiology, Faculty of Science, Chulalongkorn University, Bangkok 10330, Thailand; d Multi-Omics for Functional Products in Food, Cosmetics and Animals Research Unit, Chulalongkorn University, Bangkok 10330, Thailand; e Omics Sciences and Bioinformatics Center, Faculty of Science, Chulalongkorn University, Bangkok 10330, Thailand

**Keywords:** Gut microbiota, Microbiome, Bacteria, Feces, Type 2 diabetes, Low-calorie diet, Obesity

## Abstract

**Background:**

Dietary interventions can modulate the gut bacteria community (microbiota) and offer a complementary strategy for improving metabolic control in type 2 diabetes (T2D). This pilot study evaluated clinical clinical outcomes and gut microbiota changes following a structured low-calorie diet (LCD) intervention in obese T2D individuals under standard care.

**Methods:**

Twenty obese T2D patients were randomized into an intervention group (n = 15) (6-week 1000–1200 kcal/day of glycemic and metabolic control LCD), or a matched control group (n = 5). Clinical parameters and fecal microbiota profiles were assessed at baseline, week 6, and week 12.

**Results:**

The intervention group showed clinical trends toward improved glycemic and metabolic parameters, including reductions in fasting plasma glucose (FPG), hemoglobin A1c (HbA1c), and lipid levels (i.e., cholesterol) (*P* > 0.05), accompanied by significant loss of body weight, body mass index (BMI), and body fat (*P* < 0.05). Four intervention participants (26.7 %) achieved normoglycemia without glucose-lowering medication. Gut microbiota analyses revealed significant alterations in alpha and beta diversity over time in the intervention group (AMOVA: *P*(control baseline, intervention 12-week) = 0.025 and *P*(intervention baseline, intervention 12-week) = 0.002), with increased abundance of beneficial genera i.e. *Streptococcus*, *Bifidobacterium* and *Lactobacillus*, and enrichment of *Actinobacteria*, *Candidatus Saccharibacteria* (TM7), and *Firmicutes* at week 12. Linear discriminant analysis effect size (LEfSe) analysis identified distinct microbial biomarkers differentiating groups. Microbial functional predictions revealed significantly decreased inferred activity in pathways related to adipocytokine signaling, D-glutamine and D-glutamate metabolism, and type I diabetes mellitus (*P* < 0.05); however, these predictions were computational inferences and not experimentally validated.

**Conclusion:**

A structured LCD combined with standard care led to metabolic improvement and remodeling of gut microbiota trend in obese Thai individuals with T2D. The findings support the dietary interventions to beneficially modulate the gut microbiome and metabolic health, while highlighting the need for larger studies and functional validation.

## Graphical Abstract

**Figure:**
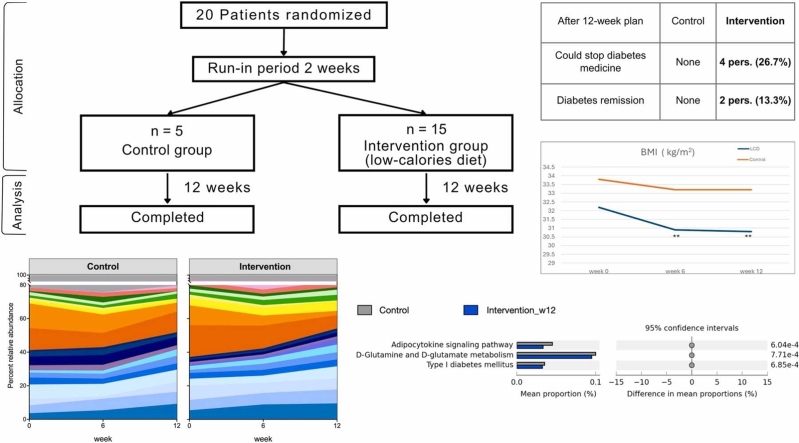

## 1 Introduction

The human gut bacteria community (microbiota) plays a central role in regulating host metabolism, immune responses, and energy homeostasis. Alterations in this microbial ecosystem—driven by diet, environment, and host factors—have been linked to metabolic disorders, including obesity and type 2 diabetes mellitus (T2D) 1, 2. Numerous microbiome studies have reported differences in gut microbiota among T2D, obesity, and healthy subjects. Dysbiosis, or disruption of balance microbial compositions, has been associated with insulin resistance, low-grade systemic inflammation, altered gut permeability, and reduced short-chain fatty acid production, all of which contribute to the pathogenesis of T2D 2, 3, 4. For examples, studies reported reductions in butyrate-producing bacteria such as *Faecalibacterium* and *Roseburia*, and increase in opportunistic pathogens, in T2D 5, 6. Such taxonomic shifts are often accompanied by altered microbial gene functions, including those related to branched-chain amino acid metabolism, lipopolysaccharide biosynthesis, short-chain fatty acid, and oxidative stress responses. These findings suggest that shift in gut microbiota could collectively contribute to glucose metabolism, insulin resistance, and mitigating T2D progression 5, 6, 7, 8.

Emerging therapeutic approaches aimed at restoring gut microbiota balance in T2D patients. Fecal microbiota transplantation (FMT) from lean, metabolically healthy donors to metabolically disordered individuals exhibited transient improvements in insulin sensitivity but faces barriers related to safety, reproducibility, and regulatory acceptance 9, 10. Alternatively, dietary intervention is a scalable and non-invasive approach with established metabolic benefits and strong influence on gut microbial compositions 11, 12, 13.

Among dietary strategies, calorie restriction and low-calorie diets (LCDs) have demonstrated consistent benefits in improving glycemic control, promoting weight loss, reducing cardiometabolic risk, and in some cases inducing T2D remission 14, 15, 16, 17, 18, 19. A landmark clinical trial (DiRECT) showed that intensive weight loss through dietary restriction could lead to T2D remission in a substantial proportion of participants 20, 21. Yet, a very limited studies on LCD intervention in successful diabetes remission in obese patients with T2D are. Meanwhile our suggested clinical successes, the extent to which LCDs affect the gut microbiota and the timepoint microbiota analysis underlying the treatment protocols have not yet been fully established and remain areas of important investigation. Some studies suggested that calorie restriction may reduce microbial diversity, while others report increased some health-associated bacteria, such as *Akkermansia*, *Bifidobacterium*, and *Lactobacillus* 22, 23, 24. No clinical studies have longitudinally assessed the gut microbiome and clinical metabolic outcomes in obese T2D patients undergoing a structured LCD intervention. Moreover, the relationship between gut microbiota changes, along their representing taxa and their microbial metabolic functions, and clinical parameter improvement (towards diabetes remission) remain non-fully clarified; and, hence, became in part the aim of this study.

Together, this pilot study evaluated the effects of a structured 6-week LCD followed by 6-week weight maintenance phase (intervention group) on gut microbiota compositions and clinical parameters compared with matched control group, in obese T2D Thai individuals receiving standard care. Through comprehensive fecal microbiota profiling, including alpha and beta diversity measures, taxonomic shifts, and computational predictions of microbial functions, we aimed to assess the interplay between dietary intervention, microbiota diversity remodeling, and metabolic health. The knowledge integration in conjunction with clinical outcomes supported the better insight into how LCD-induced microbial diversity translate into metabolic benefits, and the strategy for future more effective LCD intervention.

## 2 Materials and methods

### 2.1 Study design

**Fig. 1:**
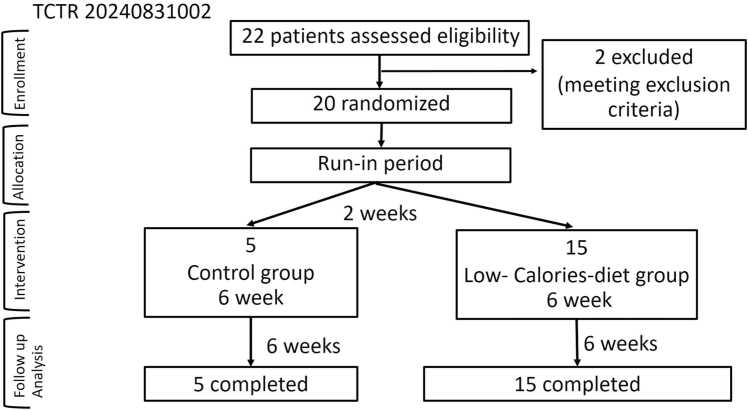
CONSORT flow diagram of 22 randomized patients (20 patients passing exclusion criteria), categorized into control group (5 patients) and intervention group (low-calorie diet, LCD) (15 patients) (TCTR abbreviates Thai Clinical Trials Registry).

This study was a prospective randomized, parallel-group clinical trial involving obese T2D patients under standard care, with and without a 12-week intervention period (intervention group vs. control group) (Fig. 1). The study design followed Consolidated Standards of Reporting Trials (CONSORT) guidelines, and the study protocol was approved by the Ethics Committee of Police General Hospital, number Dh091–66. This clinical trial was registered under the Thai Clinical Trials Registry, number 20240831002. All participants were informed about the study and provided written consent.

Patients with T2D and obesity were recruited if they had a diabetes duration of less than 10 years, a plasma HbA1c level between 6.5 % and 10 %, and a BMI greater than 23 kg/m². Patients were excluded if they had used antibiotics, probiotics, prebiotics, insulin, or GLP-1 receptor agonists within the previous 3 months. Additional exclusion criteria included serum creatinine levels > 150 μmol/L, serum ALT levels > 2.5 times the upper normal limit, inflammatory bowel disease, prior bariatric surgery, or body weight changes exceeding 5 % in the past 3 months. Sample size was calculated and a minimum total of 20 participants were sufficient to achieve 90 % statistical power to detect differences at an expected remission proportion of 0.95 [25]. In this study, we had 22 patients enrolled, and 2 patients were excluded as they failed the exclusion criteria. After a 2-week run-in period, 20 patients were randomly assigned to intervention group (n = 15) or control group (n = 5).

The clinical trial protocol consisted of three periods: (1) a 2-week run-in period where all 20 participants were introduced to a low-calorie diet for 5 days to assess compliance and dosages of glucose-lowering medications were reduced 50 % (medications were either tapered or discontinued by the endocrinologist based on individual glycemic control); (2) a 6-week low-calorie diet period where the intervention subjects consuming 1000–1200 kcal/day for 6 weeks; and (3) a 6-week weight maintenance period where the intervention subjects consuming 1400–1500 kcal/day for 6 weeks. The run-in phase was designed to minimize variability in glycemic control and medication use, allowing for a clear assessment of the intervention’s effects and also to follow the standard care for obese T2D patients. Additionally, the intervention subjects were provided 200 mL of a meal replacement product ONCE Pro (a low glycemic index medical food by Thai Otsuka Pharmaceutical Co., Ltd., Thailand) to replace one or two meals. ONCE Pro provides 14 g of fiber per 1000 kcal, which aligns with the Thai Recommended Daily Intake (RDI) of 25–38 g per day, or 14 g per 1000 kcal. Therefore, the fiber content of the formula meets national dietary guidelines and is unlikely to be the primary factor confounding the microbiota results. Both intervention and control groups received standard care, including patient education and dietary recommendations, and during each of the three study periods, participants were asked to complete dietary records to assess compliance. All participants were required to self-monitor blood glucose levels at least twice per week. To minimize recall bias and under-reporting, participants were instructed to record their meals by taking photographs of each dish. These images were reviewed by a trained dietitian, who calculated the energy and nutrient content based on standardized food composition databases. This approach provided the accurate assessment of dietary intake than traditional self-reporting methods. The medical management protocol was developed based on the Thai national guidelines.

In this study, both groups received standard care, including routine education (run-in period) and monitoring (the 6-week weight maintenance period), consistent with national clinical and ethical practice guidelines. This approach was intended to ensure ethical and equitable treatment across groups. While such support might have contributed to improvements in both groups and attenuated the observable differences attributable solely to the LCD intervention, it reflects real-world conditions where lifestyle guidance is part of standard diabetes management. All participants were instructed to perform fingerstick blood glucose measurements at least once per week, and the medical adjustment protocol, which was in accordance with the Thai national clinical guidelines, were: if the average of 2-week blood glucose readings was ≤ 140 mg/dL then glucose-lowering medications were adjusted in the following sequences (sulfonylurea (reduced or discontinued) followed by metformin); if the average of 2-week blood glucose was > 140 mg/dL then medications were reintroduced; or if the average of 2-week blood glucose was > 200 mg/dL then medications were escalated in reverse order. These medication adjustments were overseen and tailored to individual glycemic profiles by an endocrinologist. All participants were also encouraged to maintain their usual physical activity levels throughout the study period, no structured exercise program provided, and baseline dietary habits were not assessed.

ONCE Pro by Thai Otsuka Pharmaceutical Co. Ltd., had no involvement in the study design, data collection, analysis, interpretation of results, manuscript preparation, or the decision to submit for publication. The research team maintained full independence throughout the study process.

### 2.2 Clinical measurements

Blood chemistry, glycemic and metabolic parameters, body weight, and body compositions were evaluated at baseline (week 0), and weeks 6 and 12 of the intervention vs. control study period. Glycemic and metabolic parameters were measured based on an Oral Glucose Tolerance Test (OGTT), and body compositions were measured using Tanita Body Composition Analyzer SC-330 (Illinois, USA). All measurements were performed and analyzed at Police General Hospital (Bangkok, Thailand). The analysis of “could stop diabetes medicine” (if could maintain normal blood glucose level without medicine) and “diabetes remission” (if could maintain normal blood glucose level without medicine and hemoglobin A1c (HbA1c) < 6.5 %) were also evaluated from the clinical measurements by the physicians.

### 2.3 Fecal sample collections, and metagenomic DNA extraction followed by 16S rRNA gene sequencing

Stool samples were parallel collected at baseline (week 0), and weeks 6 and 12 of the intervention vs. control study period, at Police General Hospital, to analyze changes in the gut microbiota. The participants collected their stool samples after defecation and immediately handed them to the clinicians. The stool samples were stored in a −80°C freezer.

Fecal metagenomic DNA were extracted from 0.25 g of each sample using DNeasy PowerSoil Pro kit according to the manufacturer’s instructions (Qiagen, Maryland, USA). The quality and quantity of the metagenomic DNA were checked by spectrophotrometry (A260/A280 and A260) and agarose gel electrophoresis. The 16S rRNA gene variable region V3–V4 libraries were prepared by PCR using universal prokaryotic primers 341 F (5′-CCTACGGGNGGCWGCAG-3′) and 805 R (5′-GACTACHVGGGTATCTAATCC-3′) and metagenomic DNA following the 16S Metagenomic Sequencing Library Preparation Protocol (Illumina, San Diego, CA, USA) 26, 27. The final products were purified and pooled in equimolar proportion for the 2 × 250 bp next generation sequencing performed according to MiSeq Reagent Kits v2 (Illumina, San Diego, USA) and MiSeq system (Illumina) at the Omics Sciences and Bioinformatics Center, Chulalongkorn University (Bangkok, Thailand). The 16S rRNA gene sequences in this study were deposited in the NCBI open access Sequence Read Archive database (accession number PRJNA1211859).

### 2.4 Bioinformatic and statistical analyses

Raw sequences were processed following Mothur’s Standard Operating Procedure for Miseq 26, 27, 28. The forward and reverse paired-end reads were merged into single reads. Reads were filtered for quality reads based on removal of (1) short read lengths of ≤ 100 nucleotides (nt) excluding primer and barcode sequences, (2) ambiguous bases ≥ 8, (3) chimera sequences, and (4) homopolymer of ≥ 8 nt. Operational taxonomic units (OTUs) were classified at the phylum (abbreviated p_), class (c_), order (o_), family (f_), genus (g_), and species (s_) levels; and sequences of mitochondria, chloroplast, and eukaryotic lineages were removed. The alpha diversity for good’s coverage (estimate of sequencing coverage to true diversity), genus richness (number of observed OTUs, and Chao1) and genus richness and evenness estimators (Shannon, and percent relative abundances), were analyzed. Beta-diversity non-metric multidimensional scaling (NMDS) analysis at species level based on Bray–Curtis dissimilarity coefficients and Pearson’s correlation with clinical measurements were analyzed by Mothur 26, 27, 28. Linear discriminant analysis effect size (LEfSe) for microbial biomarker identification with pairwise Kruskal–Wallis and Wilcoxon tests, were analyzed following established protocols 26, 27, 28. Estimates of microbial metabolic functions were determined by PICRUSt (Phylogenetic Investigation of Communities by Reconstruction of Unobserved States) based on the reference genome annotations in KEGG (Kyoto Encyclopedia of Genes and Genomes pathways) 29, 30, 31 and statistically compared by STAMP (Statistical Analysis of Metagenomic Profiles) with two-sided Welch’s *t*-test and Storey’s FDR correction [32].

Statistical analyses were carried out using software. All data are presented as means ± standard deviations (SD) and percentages (%) for categorical outcomes. The chi-square test was used to analyze differences between groups at baseline, and ANOVA was used to detect changes in clinical parameters throughout the study period (*P* < 0.05). For microbiota and a comparison between two groups was performed by Student’s *t*-test, and comparisons among multiple parameters were performed by AMOVA (*P* < 0.05) 26, 27 unless stated. Data visualization and statistical analyses were conducted using GraphPad Prism V.10.2.3 (Graph Pad Software Inc., USA), and SPSS version 29 or R software version 3.5.2, respectively.

## 3 Results

### 3.1 Comparative clinical outcomes of patients at weeks 0, 6 and 12 following intervention vs. control study period

A total of 20 obese T2D participants entered the study, comprising 12 females and 8 males, with means of 48.1 ± 7.3 years age, 88.7 ± 19.5 kg body weight (BW) and 32.6 ± 4.9. kg/m^2^ body mass index (BMI) (Table 1). The mean duration of diabetes was 5.4 ± 3.3 years. As the control vs. intervention groups were randomly assigned, the clinical characteristics of both groups at week 0 (baseline) were similar, for examples, levels of fasting plasma glucose (FPG) (*P* = 0.916), total cholesterol (*P* = 0.591) and weight (*P* = 0.916) (Table 1). Further, of all three study periods ((1) 2-week run-in period, (2) 6-week low-calorie 1000–1200 kcal/day diet period, and (3) 6-week weight maintenance 1400–1500 kcal/day diet period), participants in both control and intervention groups showed excellence (>95 %) in self-reported dietary compliance on low-calorie intake.

**Table 1:** Baseline clinical characteristics of participants. Abbreviations include fasting plasma glucose (FPG), hemoglobin A1c (HbA1c), oral glucose tolerance test (OGTT), homeostatic model assessment of insulin resistance (HOMA-IR), quantitative insulin sensitivity check index (QUICKI), aspartate aminotransferase (AST), alanine transaminase (ALT), and body mass index (BMI).

Clinical characteristics	Control	Intervention	*P*-value
Demographics			
Age (years)	48.3 ± 2.6	41.1 ± 2.0	0.300
Male sex (%)	37.5	62.5	0.292
Duration of diabetes (years)	6.6 ± 1.9	5.2 ± 0.8	0.458
Glycemic parameters			
FPG (mg/dL)	166.2 ± 19.1	140 ± 11.0	0.916
2-h glucose after OGTT (mg/dL)	209.4 ± 33.4	173.6 ± 19.2	0.365
HbA1c(%)	7.8 ± 0.6	7.3 ± 0.3	0.434
HOMA-IR	2.9 ± 1.7	4.3 ± 1.0	0.511
QUICKI	0.3 ± 0.01	0.3 ± 0.02	0.850
Metabolic parameters			
Total cholesterol (mg/dL)	186.6 ± 19.2	174.5 ± 11.1	0.591
Triglyceride (mg/dL)	178.8 ± 37.8	129.6 ± 21.8	0.274
HDL cholesterol (mg/dL)	48.6 ± 5.1	52.2 ± 2.9	0.546
LDL cholesterol (mg/dL)	119.6 ± 17.5	102.0 ± 10.1	0.395
AST (U/L)	25.0 ± 3.5	26.9 ± 2.0	0.651
ALT (U/L)	23.4 ± 9.3	24.7 ± 5.4	0.902
Anthropometric parameters			
Weight (kg)	87.9 ± 8.9	89.0 ± 5.1	0.916
BMI (kg/m^2^)	33.8 ± 2.2	32.2 ± 1.3	0.549
%Fat	38.2 ± 4.0	39.9 ± 2.3	0.719
Fat mass (kg)	33.7 ± 5.5	35.8 ± 3.1	0.742
Fat free mass (kg)	54.1 ± 6.1	53.1 ± 3.5	0.889
Muscle mass (kg)	51.2 ± 5.9	50.1 ± 3.4	0.873
Total body water (kg)	43.1 ± 5.2	40.6 ± 3.0	0.683

Various indices of the glycemic control were found improved, either statistically or non-statistically but as consistent trends, in the intervention group since week 6, and these improvements were sustained through week 12. For instances, at weeks 6 and 12, the change from baseline for FPG and HbA1c levels in the intervention group became 130.3 ± 7.7 and 130.3 ± 7.8 mg/dL, and 6.8 ± 0.2 and 7.0 ± 0.2 % respectively, compared with in the control 167.2 ± 13.3 and 162.4 ± 13.6 mg/dL, and 7.7 ± 0.4 and 7.6 ± 0.4 % (Table 2 and Fig. S1). Yet, the glucose lowering medications were partly associated with HbA1c improvements. The 2-hour plasma glucose levels after OGTT, and other insulin resistance test as measured by HOMA-IR, were observed reduced in the intervention group (Table 2). Moreover, we found four intervention patients could withdraw the glucose-lowering medication (n = 26.7 %) and two of them achieved the HbA1c level of < 6.5 % (n = 13.3 %), vs. 0 % in control group (Table S1). Metabolic parameters including triglyceride, total cholesterol and LDL (bad) cholesterol were reduced, and HDL (good) cholesterol was relatively increased, in the intervention (Table 2).

**Table 2:** Effects of low-calorie diet on various clinical parameters and mean changes at weeks 6 and 12. Abbreviations include fasting plasma glucose (FPG), oral glucose tolerance test (OGTT), hemoglobin A1c (HbA1c), homeostatic model assessment of insulin resistance (HOMA-IR), quantitative insulin sensitivity check index (QUICKI), aspartate aminotransferase (AST), alanine transaminase (ALT), and body mass index (BMI). Mean difference ± SEM and *P*-value by time at week 6 inferred the comparison of week 0 to week 6 of the same corresponding group, and mean difference ± SEM and *P*-value by time at week 12 inferred the comparison of week 0 to week 12 of the same corresponding group; so no (or positive) sign in front of mean difference ± SEM represents the reduction in that clinical parameter, and minus sign represents the increase in that clinical parameter.

Variable	Week 6				Week 12			
	Control		Intervention		control		Intervention	
	Mean ± SEM		Mean ± SEM		Mean ± SEM		Mean ± SEM	
	Mean difference ± SEM	*P*-value by time	Mean difference ± SEM	*P*-value by time	Mean difference ± SEM	*P*-value by time	Mean difference ± SEM	*P*-value by time
Glycemic control indices								
FPG (mg/dL)	167.2 ± 13.3		130.3 ± 7.7		162.4 ± 13.6		130.3 ± 7.8	
	1.0 ± 13.5	0.942	9.7 ± 7.8	0.230	3.8 ± 17.9	0.834	9.7 ± 10.3	0.361
2-h glucose after OGTT (mg/dL)	216.0 ± 32.6		165.8 ± 18.8		218.0 ± 33.2		185.6 ± 19.2	
	-6.6 ± 28.3	0.818	7.8 ± 16.4	0.639	-8.6 ± 24.0	0.725	-12.0 ± 13.9	0.399
HbA1c (%)	7.7 ± 0.4		6.8 ± 0.2		7.6 ± 0.4		7.0 ± 0.2	
	0.1 ± 0.4	0.760	0.4 ± 0.2	0.080	0.2 ± 0.4	0.655	0.3 ± 0.2	0.247
HOMA-IR	3.8 ± 1.0		3.3 ± 0.6		4.4 ± 1.3		3.5 ± 0.7	
	-0.9 ± 1.3	0.501	0.9 ± 0.8	0.245	-1.5 ± 1.4	0.289	0.8 ± 0.8	0.320
QUICKI	0.7 ± 0.16		0.3 ± 0.09		0.32 ± 0.02		0.3 ± 0.01	
	-0.33 ± 0.16	0.056	-0.00 ± 0.09	0.977	0.01 ± 0.01	0.504	-0.00 ± 0.01	0.698
Metabolic parameters								
Total cholesterol (mg/dL)	162.8 ± 17.9		158.8 ± 10.3		185.6 ± 18.0		174.5 ± 10.4	
	23.8 ± 21.7	0.287	15.7 ± 12.5	0.227	1.0 ± 17.1	0.954	-0.7 ± 9.9	0.995
Triglyceride (mg/dL)	125.2 ± 25.7		120.7 ± 14.8		136.4 ± 27.7		123.1 ± 16.0	
	53.6 ± 26.2	0.057	8.9 ± 15.1	0.567	42.4 ± 22.2	0.072	6.5 ± 12.8	0.617
HDL cholesterol (mg/dL)	45.6 ± 4.6		50.8 ± 2.6		50.6 ± 4.6		52.9 ± 2.7	
	3.0 ± 3.1	0.342	1.4 ± 1.8	0.441	-2.0 ± 2.9	0.494	-0.7 ± 1.7	0.633
LDL cholesterol (mg/dL)	105.2 ± 17.8		89.1 ± 10.3		122.4 ± 20.5		105.3 ± 11.2	
	14.4 ± 16.9	0.405	12.9 ± 9.8	0.201	-2.8 ± 15.3	0.857	-3.7 ± 8.9	0.716
AST (U/L)	20.0 ± 3.2		25.2 ± 1.9		26.2 ± 4.2		27.9 ± 2.5	
	5.0 ± 2.8	0.088	1.7 ± 1.6	0.312	-1.2 ± 4.6	0.799	-1.0 ± 2.7	0.714
ALT (U/L)	19.4 ± 8.1		23.5 ± 4.7		21.0 ± 7.8		24.4 ± 4.5	
	4.0 ± 4.8	0.419	1.3 ± 2.8	0.656	2.4 ± 5.1	0.645	0.3 ± 3.0	0.912
Anthropometric parameters								
Weight (kg)	86.6 ± 8.4		85.1 ± 4.9		86.5 ± 8.3		84.7 ± 4.8	
	1.2 ± 1.3	0.361	3.8 ± 0.8	**< 0.001**	1.4 ± 1.6	0.396	4.2 ± 0.9	**< 0.001**
BMI (kg/m^2^)	33.2 ± 2.2		30.9 ± 1.3		33.2 ± 2.2		30.8 ± 1.2	
	0.6 ± 0.4	0.192	1.3 ± 0.2	**< 0.001**	0.6 ± 0.5	0.320	1.4 ± 0.3	**< 0.001**
%Fat	37.5 ± 4.0		38.1 ± 2.3		37.7 ± 4.1		37.7 ± 2.4	
	0.8 ± 0.6	0.246	1.8 ± 0.4	**< 0.001**	0.5 ± 0.9	0.535	2.2 ± 0.5	**< 0.001**
Fat mass (kg)	32.6 ± 5.1		7. ± 2.9		32.7 ± 5.1		32.3 ± 2.9	
	1.1 ± 1.1	0.303	3.1 ± 0.6	**< 0.001**	1.1 ± 1.3	0.428	3.5 ± 0.8	**< 0.001**
Fat free mass (kg)	54.0 ± 5.9		52.4 ± 3.4		53.8 ± 5.9		52.4 ± 3.4	
	0.1 ± 0.4	0.826	0.74 ± 0.2	**0.002**	0.3 ± 0.4	0.434	0.68 ± 0.2	**0.012**
Muscle mass (kg)	51.1 ± 5.7		49.4 ± 3.3		50.9 ± 5.7		49.4 ± 3.3	
	0.1 ± 0.3	0.817	0.7 ± 0.2	**0.003**	0.3 ± 0.4	0.431	0.6 ± 0.2	**0.012**

While both intervention and control groups showed a decrease in body weight, the intervention revealed relatively more reduced body weight and BMI: week 6, intervention group decreased body weight by averagely −3.8 ± 0.8 kg and control group −1.2 ± 1.3 kg; and week 12 was intervention −4.2 ± 0.9 kg and control −1.4 ± 1.6 kg. For BMI, the intervention decreased by averagely double of the control group (Table 2).

Nonetheless, analyses of the dietary records revealed the patients’ substantial reduction in calorie intake in both groups, provided that the total energy intake was 1002.2 ± 258.8 kcal/day in the control group and 995.5 ± 124.8 kcal/day in the intervention group (no statistical differences in self-reported dietary compliances between groups). Additionally, no significant differences in parameters related to the consumption of micronutrients or antioxidants, was detected between groups. Hence, during the final period of weight maintenance of the intervention group where the intervention participants resumed calorie/day amount, both control and intervention group participants reported similar dietary intake (data not shown). Additionally, no significant differences in parameters related to the consumption of micronutrients or antioxidants, were detected between groups.

### 3.2 Comparative gut microbiota and potential microbial metabolic profiles of intervention vs. control patients at baseline and weeks 6 and 12 study period

**Fig. 2:**
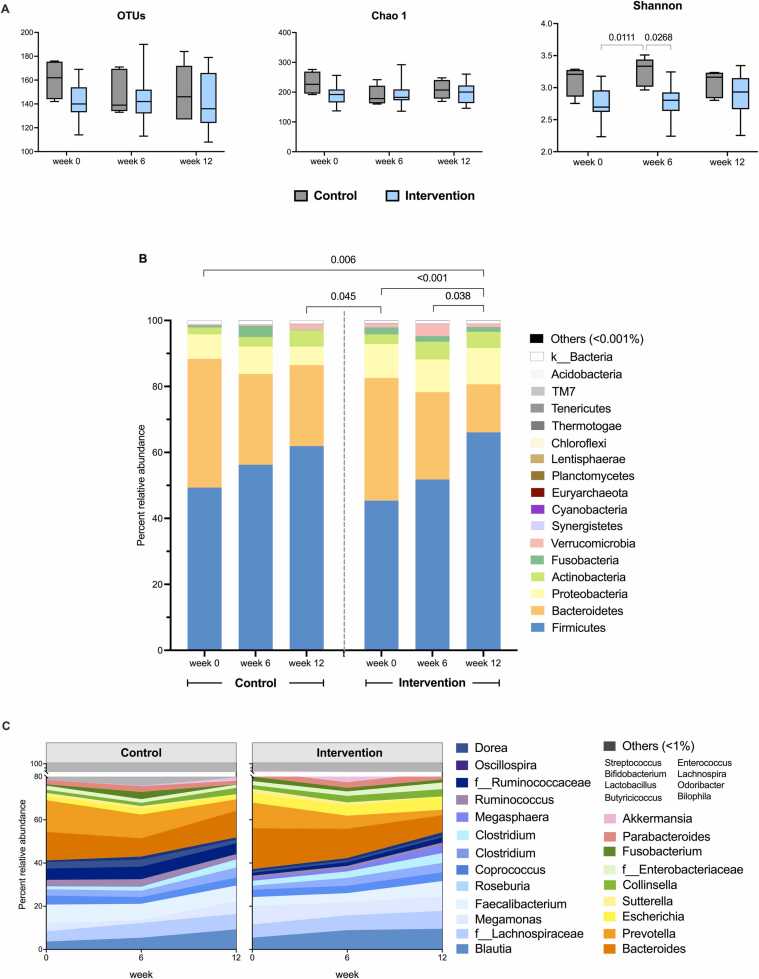
(A) Alpha-diversity analysis at genus level including operational taxonomic unit (OTUs), Chao1 and Shannon indices; and microbiota compositions in percent relative abundance at (B) phylum and (C) genus level OTUs (AMOVA, *P* < 0.05). In (C), OTUs were classified to the deepest taxonomic level where allowed: f_ abbreviates family.

Gut microbiota of intervention and control patients were compared through the study period (weeks 0, 6 and 12), and that the sequencing coverages of all samples were successful yielding > 99 % Good’s coverage (Table S2). Alpha-diversity analysis at genus level revealed decrease in diversity in intervention week 6 (Fig. 2 A: *P* = 0.0268). Bacterial phylum compositions revealed statistical differences in intervention week 6 from week 12 (Fig. 2B: *P* = 0.038) and baseline (*P* < 0.001), and between the control and intervention (Table 3: *P*(control week 0, intervention week 12) = 0.025 and *P*(intervention week 0, intervention week 12) = 0.002). Phyla Firmicutes, Bacteroidetes, Proteobacteria, Actinobacteria and Fusobacteria (or Verrucomicrobia) accounted for core bacteria among groups. Compared with the control group, undergoing intervention (week 6) exhibited relatively increase of Actinobacteria and Verrucomicrobia, and reduced Fusobacteria (Fig. 2B). Analysis in bacterial genera compositions further showed that intervention week 6 comprised unique bacterial community structure, e.g. relatively increase *Akkermansia, Collinsella*, *Clostridium, Megamonas* and *Blautia*, and decrease of *Oscillospira, Ruminococcus*, *Prevotella* (Fig. 2 C).

**Table 3:** AMOVA statistics of gut microbiome compositions at species OTUs between sample groups.

**Control**	**Intervention**	**p-value**
Week 0	Week 0	0.243
	Week 6	0.189
	Week 12	0.025*
**Intervention**	**Intervention**	**p-value**
Week 0	Week 6	0.293
	Week 12	0.002*
Week 6	Week 0	0.453

**Fig. 3:**
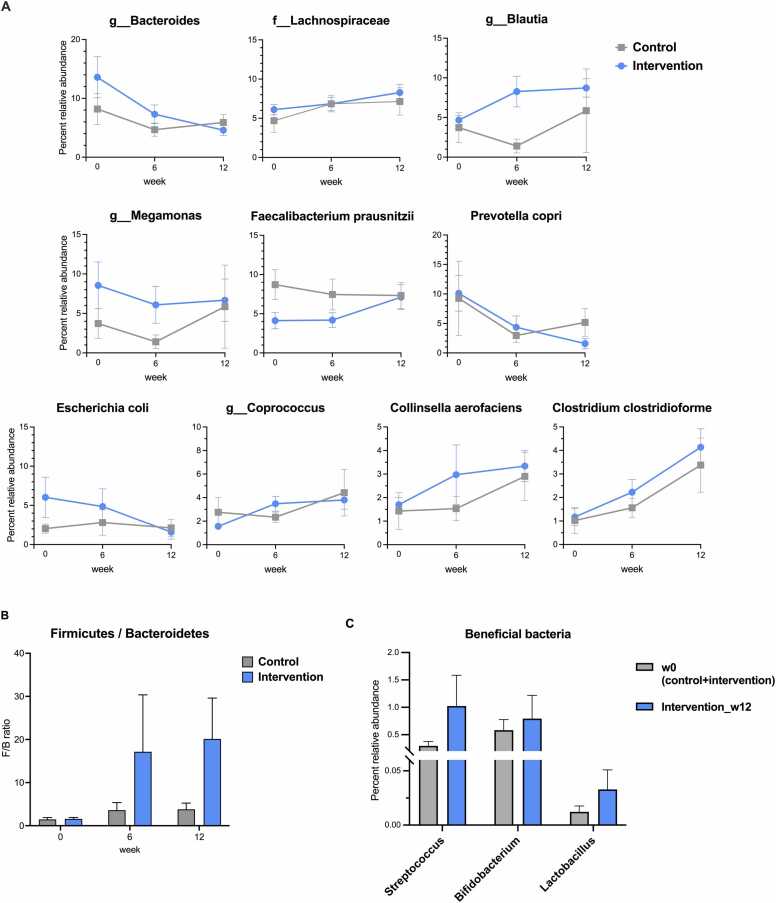
(A) Percent relative abundance of top 10 most abundance species, (B) Firmicutes-to-Bacteroidetes ratio, and (C) beneficial bacterial genus in gut (*Streptococcus*, *Bifidobacterium* and *Lactobacillus*). In (A), OTUs were classified to the deepest taxonomic level where allowed: f_ abbreviates family; and g_, genus.

**Fig. 4:**
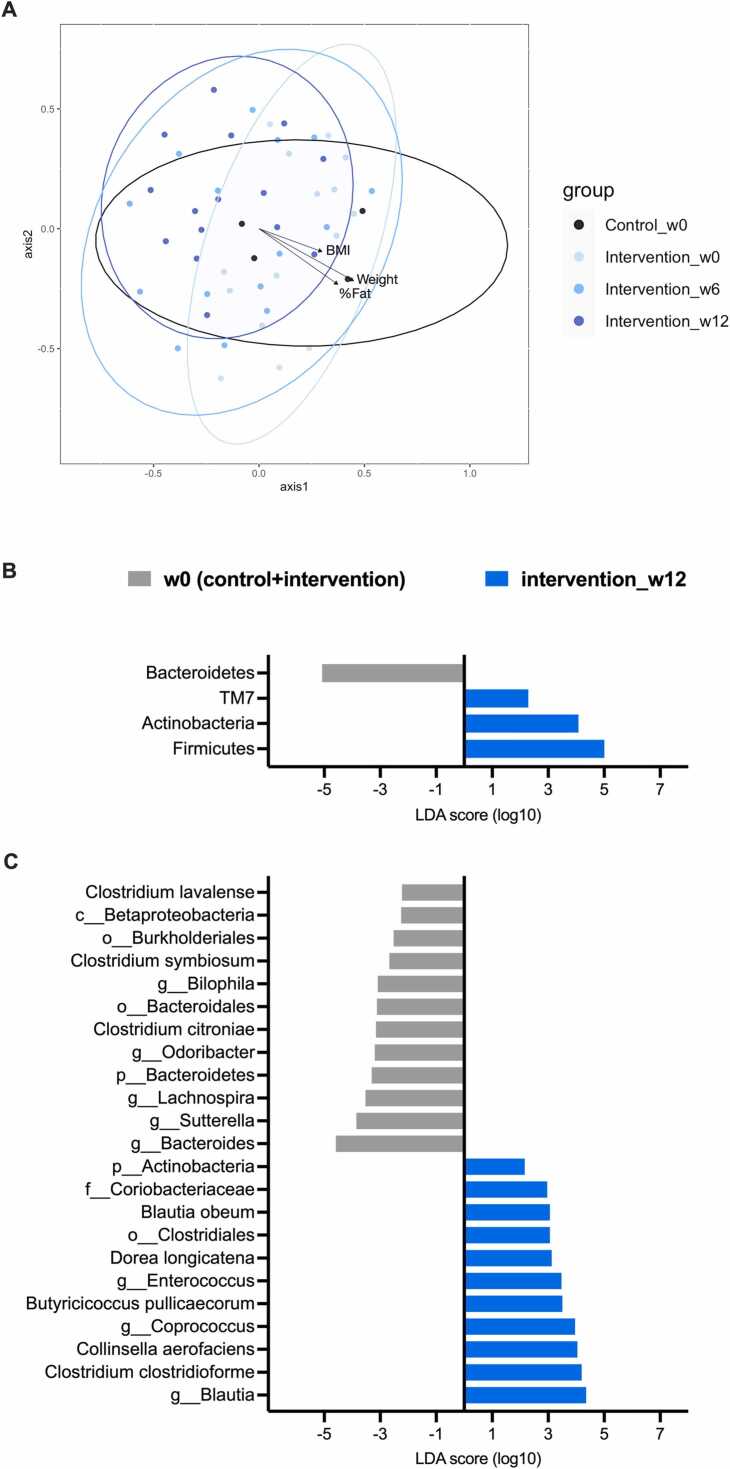
(A) Non-metric multidimensional scaling (NMDS) based on the Bray–Curtis dissimilarity index and Pearson’s correlation with weight, BMI and fat (*P* < 0.05); and LEfSe analysis of week 0 vs. intervention week 12 at (B) phylum and (C) species levels (linear discriminant analysis, or LDA score, > 2.0 indicates statistical difference). In (C), OTUs were classified to the deepest taxonomic level where allowed: p_ abbreviates phylum; c_, class; o_, order; f_, family; and g_, genus.

The topmost abundant species were compared, and focusing after intervention we found that the week 6 control and intervention groups showed differences in *Blautia*, *Megamonas, Collinsella aerofaciens*, and *Faecalibacterium prausnitzii* (Fig. 3 A: *P* > 0.05). The Firmicutes-to-Bacteroidetes ratio (F/B) (generally the F/B ratio should be ∼1.0 to represent a balance in gut health) 33, 34, 35 showed increase after intervention, yet non-statistically significant (Fig. 3B: *P* > 0.05). Beneficial intestine bacterial genera *Streptococcus*, *Bifidobacterium* and *Lactobacillus* were all found increased after the intervention (Fig. 3 C: *P* > 0.05). Supportively, the beta-diversity analysis for microbiota structure comparisons among groups showed the relative diverse microbiota structure after the interventions, and the intervention microbiota structures (Fig. 4 A: intervention_w6 and intervention_w12) had statistical negative Pearson’s correlation with weight, BMI and body fat.

The LEfSe analysis was included to determine phylum and species biomarkers of the baseline (control and intervention) vs. intervention group. Consistent with the aforementioned bacterial composition analyses, for examples, Actinobacteria and *Blautia* remained biomarker for the intervention (Figs. 4B and 4 C). Comparing microbiota between the control and the particular diabetes remission in intervention highlighted species markers of *Fusobacterium*, *Streptococcus*, *Ruminococcus bromii*, *Clostridium celatum* (in family Lachnospiraceae) (another SCFA-producing bacteria) and *Bifidobacterium longum* (in phylum Actinobacteria) (Fig. S2).

**Fig. 5:**
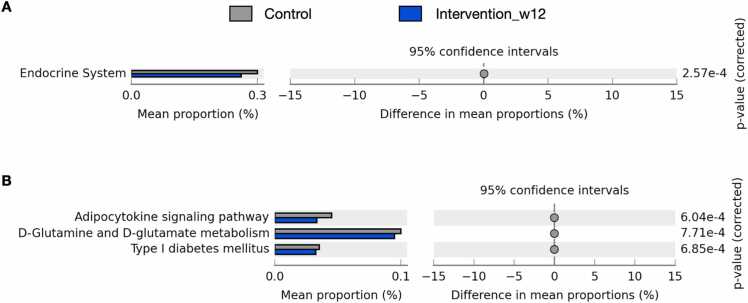
Comparative predicted microbial metabolic profiles between control and intervention week 12 in KEGG pathways (A) level 2 and (B) level 3 (Welsh’s *t*-test, *P* < 0.001). Noted that only statistically different pathways that involve human system were displayed.

The potential microbial metabolic profiles of the control vs. the intervention at week 12 were compared, and the functions involved endocrine system, adipocytokine signaling pathway, D-glutamine and D-glutamate metabolism, and type 1 diabetes mellitus showed statistically higher in the control than the 12-week intervention groups (*P* < 0.001) (Fig. 5). The finding was consistent with the aforementioned clinical data records and the success in intervention individuals who achieved diabetes remission (Fig. 2 C, 3 C, and S2).

Multivariate correlation analysis demonstrated that the intervention significantly reshaped the overall associations between microbial taxa, predicted microbial metabolic potentials, and clinical parameters (permutation ANOVA, *P* = 0.011). The intervention_w12 samples formed a distinct cluster separated from the intervention_w0 baseline, indicating a sustained intervention-driven shift in the microbiota–clinical relationship. This new profile was characterized by higher relative abundance of beneficial taxa—including *Akkermansia muciniphila* and *Clostridium hiranonis*—together with lower inferred activity of pathways such as D-glutamine and D-glutamate metabolism. In contrast, the control samples were associated with genera such as *Oscillospira* and *Megasphaera*, along with clinical markers including body weight and cytokine signaling activity. These data supportively highlight distinct and divergent microbiota–clinical associations between the intervention and control groups (Fig. S3).

## 4 Discussion

To our knowledge, this is the first randomized clinical prospective cohort trial to provide evidence for the efficacy of a structured low-calorie diet, in combination with standard diabetes care, that can induce clinically meaningful metabolic improvements and modulate gut microbiota composition in obese Thai individuals with type 2 diabetes (T2D). Several studies have demonstrated that modulating gut microbiota through various dietary patterns can help control T2D; however, these associations vary, as people generally consume mixed foods rather than adhering to specifically prescribed dietary patterns alone 2, 11, 12. In this study, we compared the effects of a 6-week low-calorie diet intervention with a control condition in 20 individuals with obesity and T2D, all under standard care, for over a 12-week period.

While it might be expected that a reduction in calorie intake could influence and deplete some gut microbial richness 16, 36, our findings supported the evidence and yet reported the unique bacterial genera and microbiota pattern following the LCD intervention, in which the changes might be associated with the improved clinical data findings and the successful diabetes remission in the intervention group. Four of 15 intervention participants (26.7 %) were consistent with the remission rates reported in large-scale lifestyle intervention trials. In the Look AHEAD study, which enrolled over 5000 individuals with T2D, remission was achieved in a modest proportion of participants and was often not sustained long-term (11.5 % in first year and 7.3 % after follow up 4 year). Importantly, even partial or temporary remission in Look AHEAD was associated with significant reductions in cardiovascular and kidney disease risk [37]. Our findings, though limited by sample size and study duration, suggested clinically meaningful improvements and support the potential of dietary interventions in promoting metabolic health in obese T2D Thai individuals. The positive clinical signs against obesity and/or T2D, for instances, FPG, HbA1c, insulin resistance assessment and cholesterol, were reported. Overall, the LCD patients demonstrated consistent trends toward improvement in several clinical parameters across timepoints, and also demonstrated positive changes in the beneficial intestinal bacteria associated with diabetes, including *Streptococcus*, *Bifidobacterium* and *Lactobacillus* 16, 38, 39, 40, 41, 42. In details, many species in *Lactobacillus* can help ameliorate insulin resistance by increasing glucose transporter type 4 (GLUT-4), lipid phosphatidylinositol-3-kinase (PI3K), insulin receptor substrate 2 (IRS2), energy sensor adenosine monophosphate protein kinase (AMPK), and blood glucose hormone glucagon synthesis expressions; and provide potential anti-diabetic effect 16, 42. *Bifidobacterium* ameliorates fat accumulation, increases insulin sensitivity, and enhances glycogen synthesis (a process in which glucose is stored as glycogen in liver or muscle cells for later use) via producing bile salt hydrolases that convert bile salts to deconjugated or secondary bile acids. Then, secondary bile acids can activate the membrane bile acid receptor (i.e., TGR5) and induces the production of hormone glucagon-like peptide-1 (GLP-1, a gut hormone that stimulates insulin secretion and plays a key role in glucose homeostasis, appetite regulation, and gastrointestinal motility 43, 44. *Bifidobacterium longum* helps combat obesity and T2D by these enhanced glucose and lipid metabolisms, appetite and energy balance mechanisms, as well as the SCFAs acetate and butyrate productions that impact the tight intestinal cell junction, suppress systemic inflammation that could contribute to insulin, and restore gut microbial homeostasis such as *Akkermansia*, in replace of enteric pathobionts such as Enterobacteriaceae 45, 46.

However, our study was partly biased by the factor that both control and intervention patients responded well to the 2-week run-in period and self-dietary compliances that resulted in significant lower calorie intake for both groups. This could interference the clear distinction by the intervention. Additionally, the LCD intervention diet formula might limit the fiber intake compared with the normal diet in the control. For examples, a high in the F/B ratio and *Collinsella*, which both were previously reportedly related to low-fiber diet, inflammation, and could support obesity, T2D or elevated cholesterol 14, 47, 48, 49, 50, 51. The significantly increased Firmicutes and thus the F/B ratio, similar to our report. Still, these are inconsistency in the reported associations between the F/B ratio and T2D [30]. By the way, our protocols that contain similarity in final 6-week follow up where the reported calorie intake between the intervention and control groups during the weight maintenance phase, while the protocols aimed to preserve the metabolic and microbiota improvements achieved during the intervention. We acknowledged that this might attenuate observable differences in outcomes.

Our findings highlighted that the patients could maintain the LCD intervention (through 12 weeks). At 12-week intervention in the individuals who achieved diabetes remission exhibited representing bacterial biomarkers of, for examples, *C. celatum* and *B. longum*. The *C. celatum* is known for SCFA production, especially butyrate, and play roles in host lipid signaling, gut barrier, and gut health (e.g., against inflammatory bowel syndrome) 50, 51, 52, 53. Finally, the microbial metabolic profiles were estimated and supported the microbiota findings, in aspects of beneficial bacteria and the analyzed beneficial pathways (statistically lower in endocrine system, adipocytokine signaling pathway, D-glutamine and D-glutamate metabolism, and type 1 diabetes mellitus) in the intervention group. Still, the 12-week period might not be sufficient to fully assess the long-term effects or sustainability of the intervention, although the 12-week study period was sufficient to evaluate the initial efficacy and short-term metabolic impact given that the 12-week timeframe was selected based on the prior evidence demonstrating significant glycemic improvements and remission potential within this period 17, 25.

Meanwhile this study served to support the 6-week LCD intervention could show clinically with supportively gut microbiome changes for the obese with T2D patients, this study had limitations. First, the relatively small sample size and single-center design may limit generalizability and reduce statistical power. The small control group (N = 5) might limit robustness and increase the risk of random error or outlier impact, and we acknowledged the need for future larger and balanced group sizes. Even though the Police General Hospital is a tertiary care center that receives referrals from across Thailand, the study population likely reflects a range of genetic backgrounds and regional diversity. We acknowledged that the results may not be representative of other populations or genetic backgrounds, and highlight the need for multi-center studies in diverse settings to confirm and extend our findings. Second, although dietary intake was closely monitored and self-reported compliance was high, the significant calorie reduction in both the control and intervention groups could partially obscure the intervention effect. Additionally, the low fiber content (i.e., the LCD formula) might have contributed to microbiota shifts such as increased F/B ratio and enrichment of *Collinsella*, which have previously been linked to low-fiber intake and metabolic dysfunction. Nonetheless, the intervention group showed distinct improvements in metabolic markers, gut microbial diversity, and the enrichment of several taxa that are known for SCFA production, anti-inflammatory and metabolic, and anti-T2D benefits. Functional predictions of the microbiome also aligned with the observed clinical improvements, indicating reduced activation of pathways associated with inflammation and diabetes. Kindly noted that the microbial functional inferences were from the 16S rRNA gene data, so remained the predictive estimates and validation via shotgun metagenomic microbial functional profiles and/or experimental validation may be appropriate. Together, the results supported the larger-scale (larger sample size), longer intervention period, multi-center studies, and baseline dietary assessments of individuals, to confirm the efficiency and mechanistic underlie of LCD-induced observable trends in gut microbiota composition remodeling in obese with T2D patients in Thai genetics.

## 5 Conclusions

This pilot study suggested that a structured low-calorie diet, when combined with standard care, could potentially beneficially alter the gut microbiota compositions and metabolic functions in obese patients with T2D, which contributed to clinical improvements in glycemic control and weight loss. These observable trends in microbiota shifts, including increased abundance of SCFA-producing and anti-inflammatory taxa, were associated with the clinical improvements and diabetes medicine remission in some patients. While the long-term and system biology (combined microbiota and metabolic marker study) effects remain to be determined, this study supported the potential of dietary intervention as a non-pharmacological strategy to modulate host–microbiota interactions for improved metabolic health. Future research may focus on development of optimized dietary and personalized dietary-microbiome strategies to achieve more successful diabetes remission treatment outcome in obese with T2D individuals, including perhaps use of isocaloric non-restrictive or blinded dietary controls.

## CRediT authorship contribution statement

**Naraporn Somboonna:** Writing – review & editing, Writing – original draft, Visualization, Validation, Supervision, Resources, Project administration, Methodology, Investigation, Funding acquisition, Formal analysis, Data curation, Conceptualization. **Thanya Cheibchalard:** Visualization, Validation, Methodology, Investigation, Formal analysis, Data curation. **Mongkontida Umphonsathien:** Writing – review & editing, Writing – original draft, Validation, Resources, Project administration, Methodology, Investigation, Funding acquisition, Formal analysis, Data curation, Conceptualization. **Pornsawan Prutanopajai:** Validation, Methodology, Investigation, Formal analysis.

## Declaration of Competing Interest

The authors declare that they have no known competing financial interests or personal relationships that could have appeared to influence the study reported in this paper. The authors have no conflict of interest.

## Data Availability

The 16S rRNA gene sequences in this study were deposited in the NCBI open access Sequence Read Archive database (accession number PRJNA1211859).
