# Supplementary material for: Low-calorie diet intervention ameliorates gut microbiota dysbiosis and metabolic changes in obese patients with type 2 diabetes under standard care
Source: Comput Struct Biotechnol J. 2025 Nov 20;27:5307–17. doi: 10.1016/j.csbj.2025.11.043 (PMC12686632; doi:10.1016/j.csbj.2025.11.043)
Supplement: Supplementary file 1 — Supplementary material [file mmc1.docx]

**Supporting information**


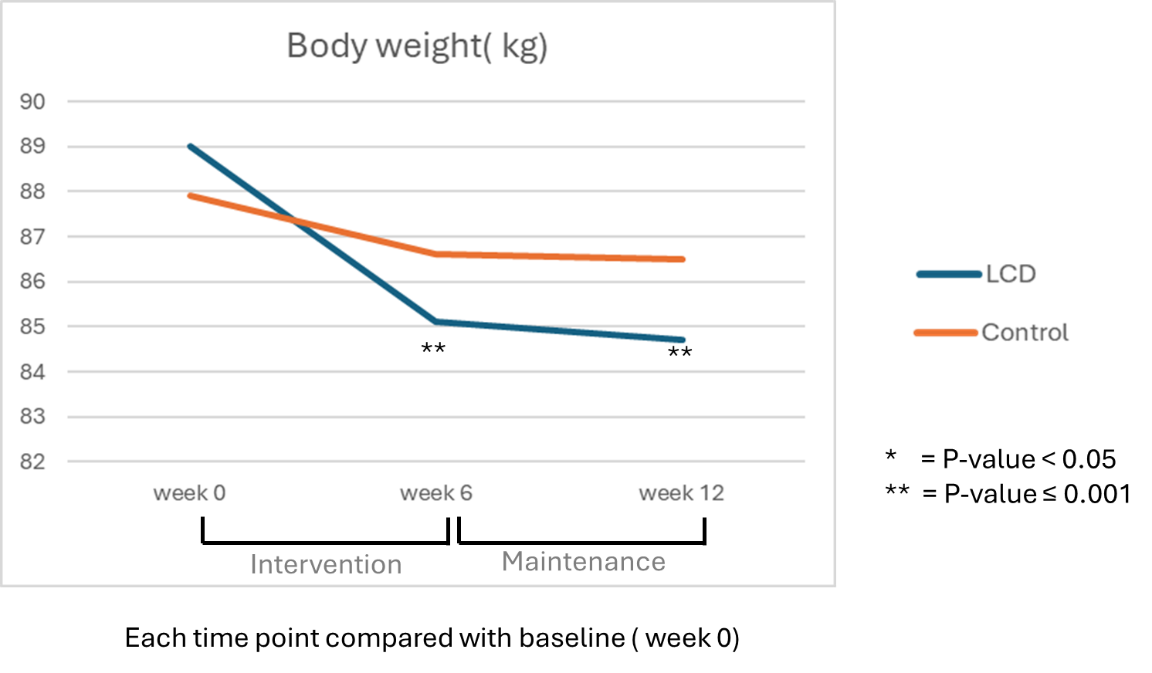


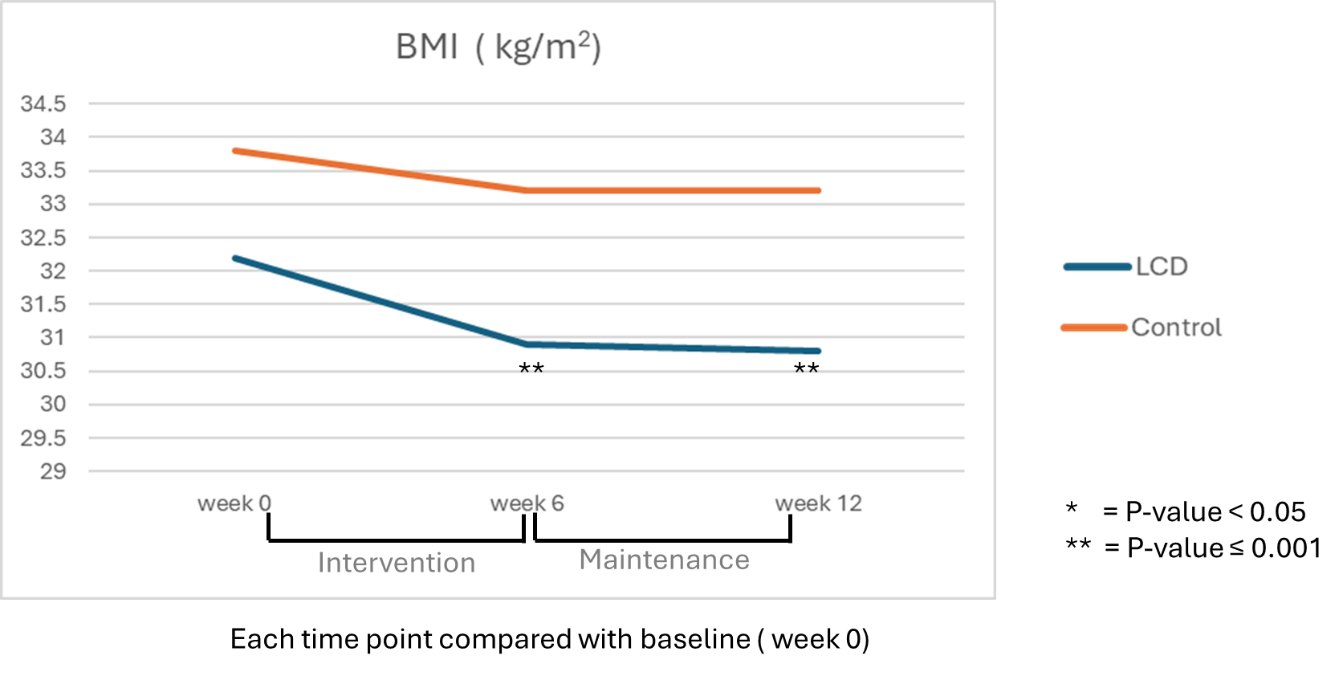


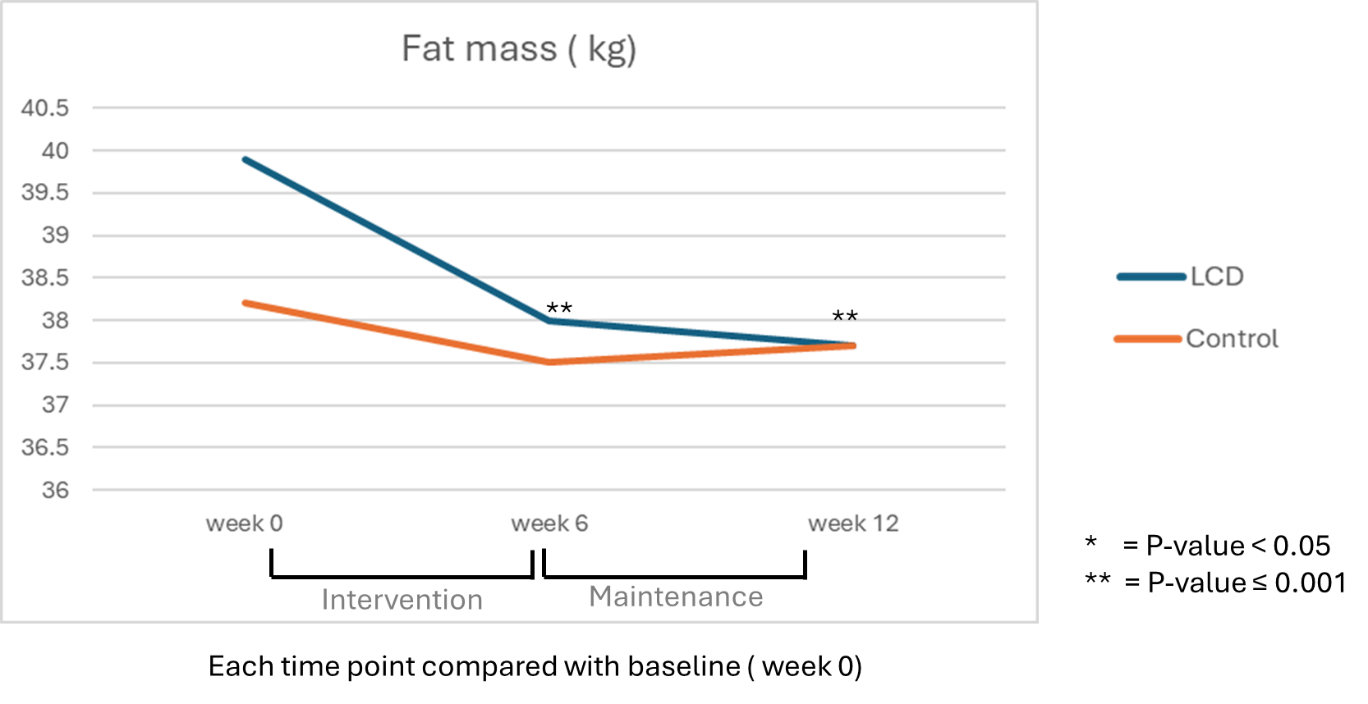


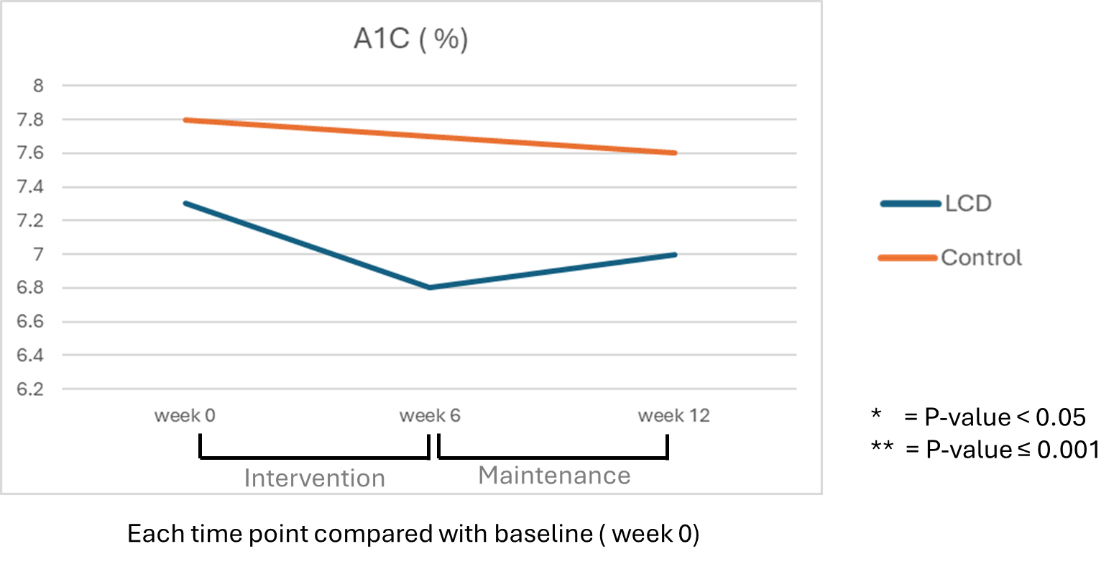


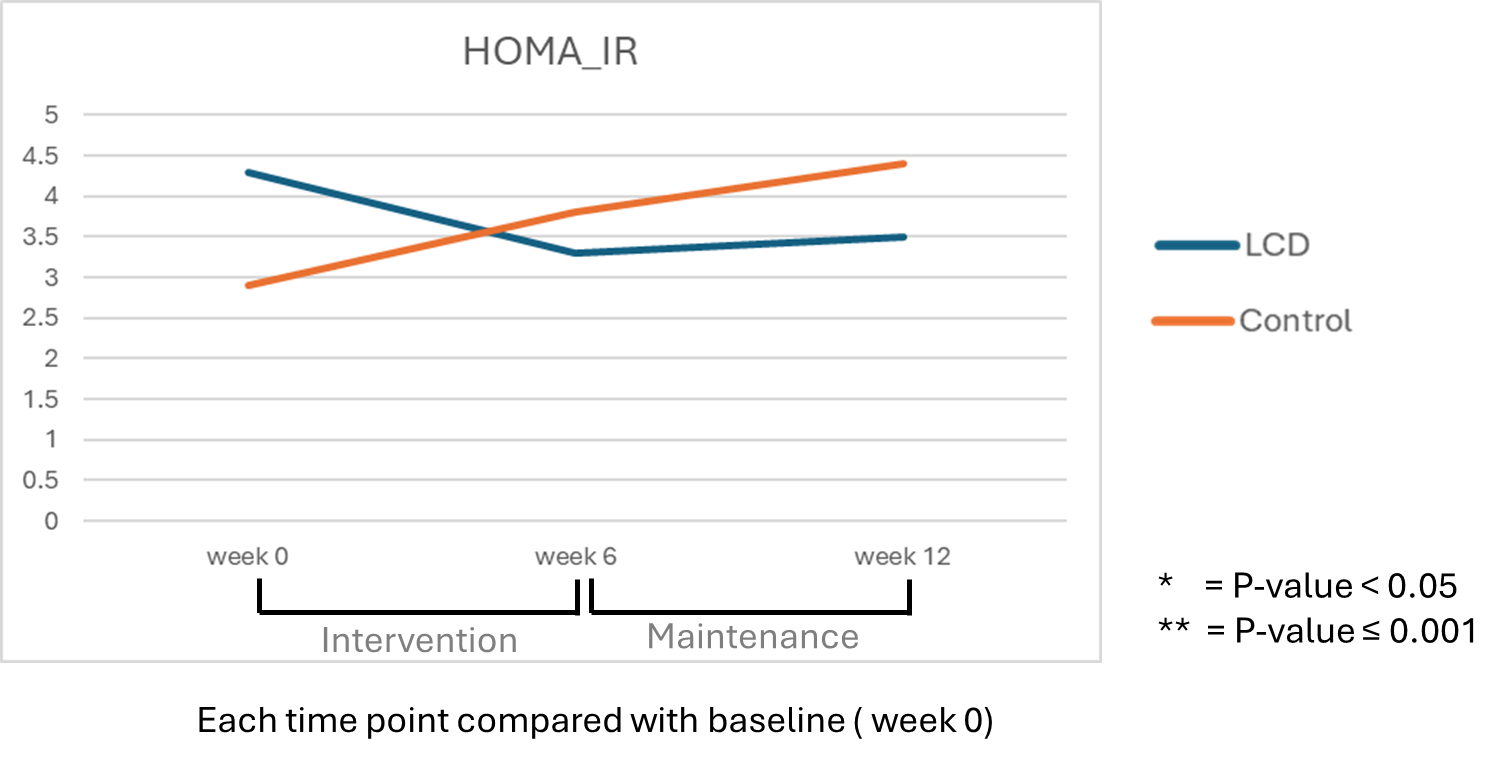


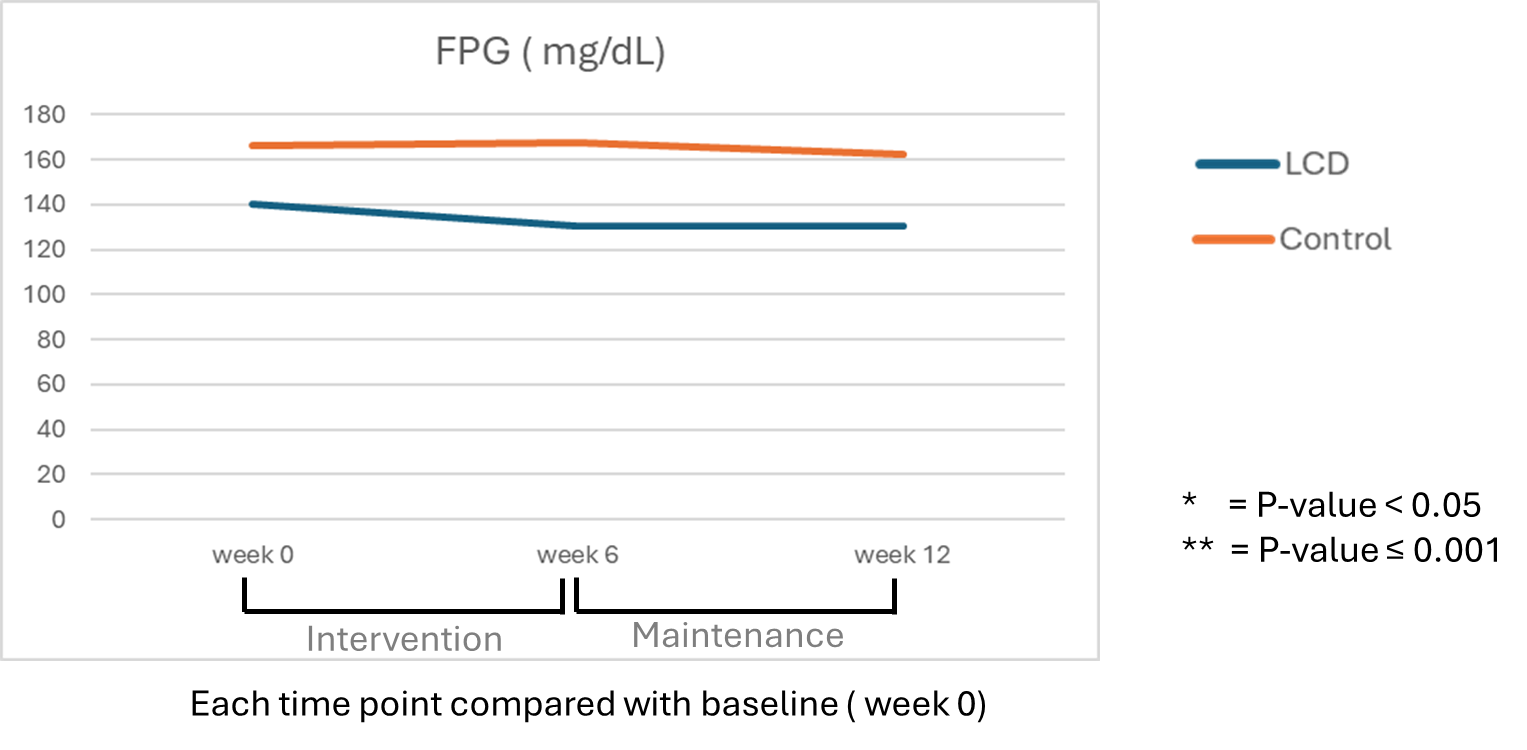


**Fig. S1.** Selected key clinical numeric differences following LCD intercention vs. control groups. Abbreviations include hemoglobin A1c (HbA1c or A1c), homeostatic model assessment of insulin resistance (HOMA-IR), and fasting plasma glucose (FPG).


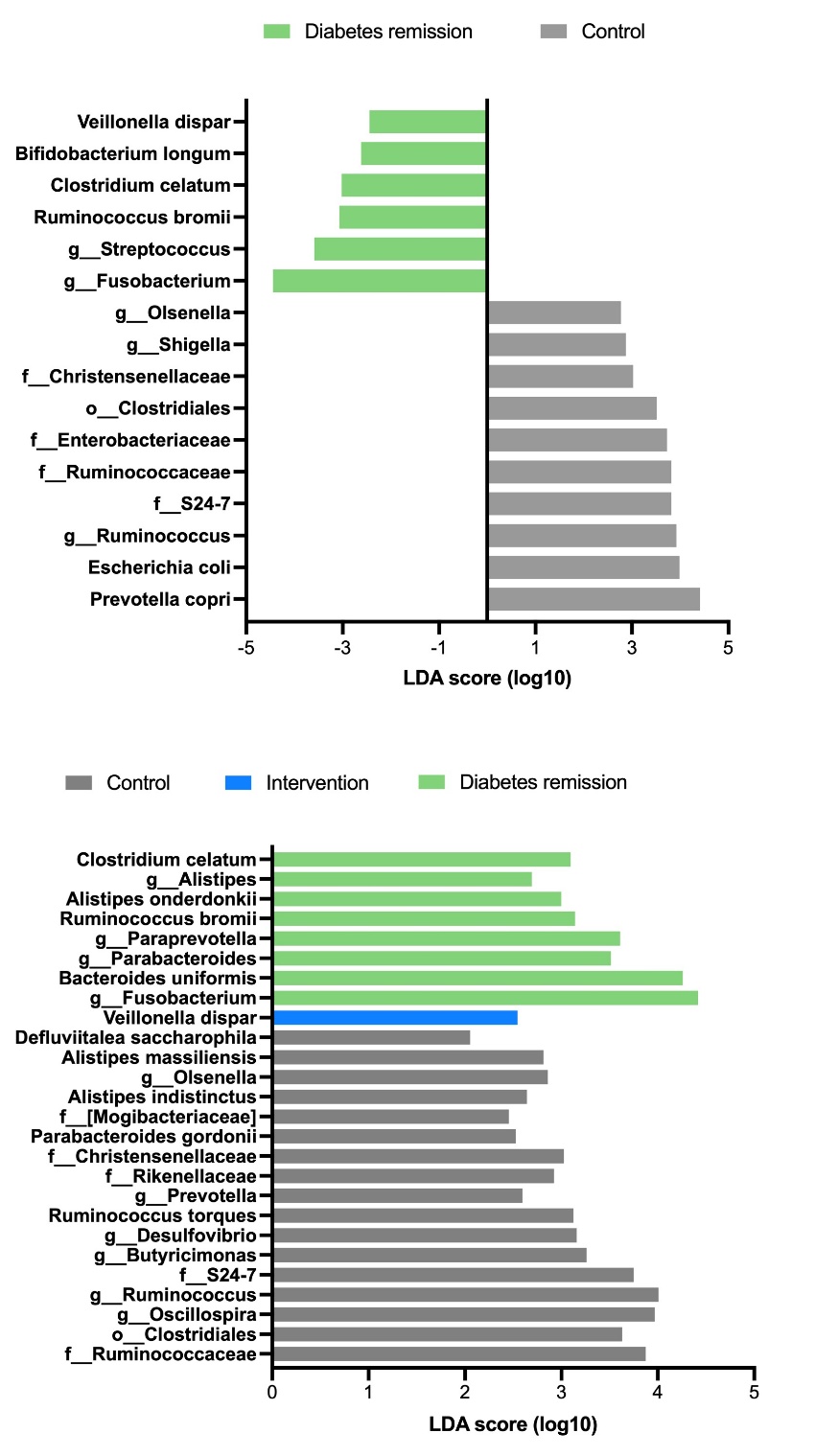


**Fig. S2.** LEfSe analysis at microbiota species level (LDA score > 2.0 indicates statistical difference) between control and diabetes remission groups, and among control vs. intervention vs. diabetes remission groups. The OTUs were classified to the deepest taxonomic level where allowed: o_ abbreviates order; f_, family; and g_, genus.

**
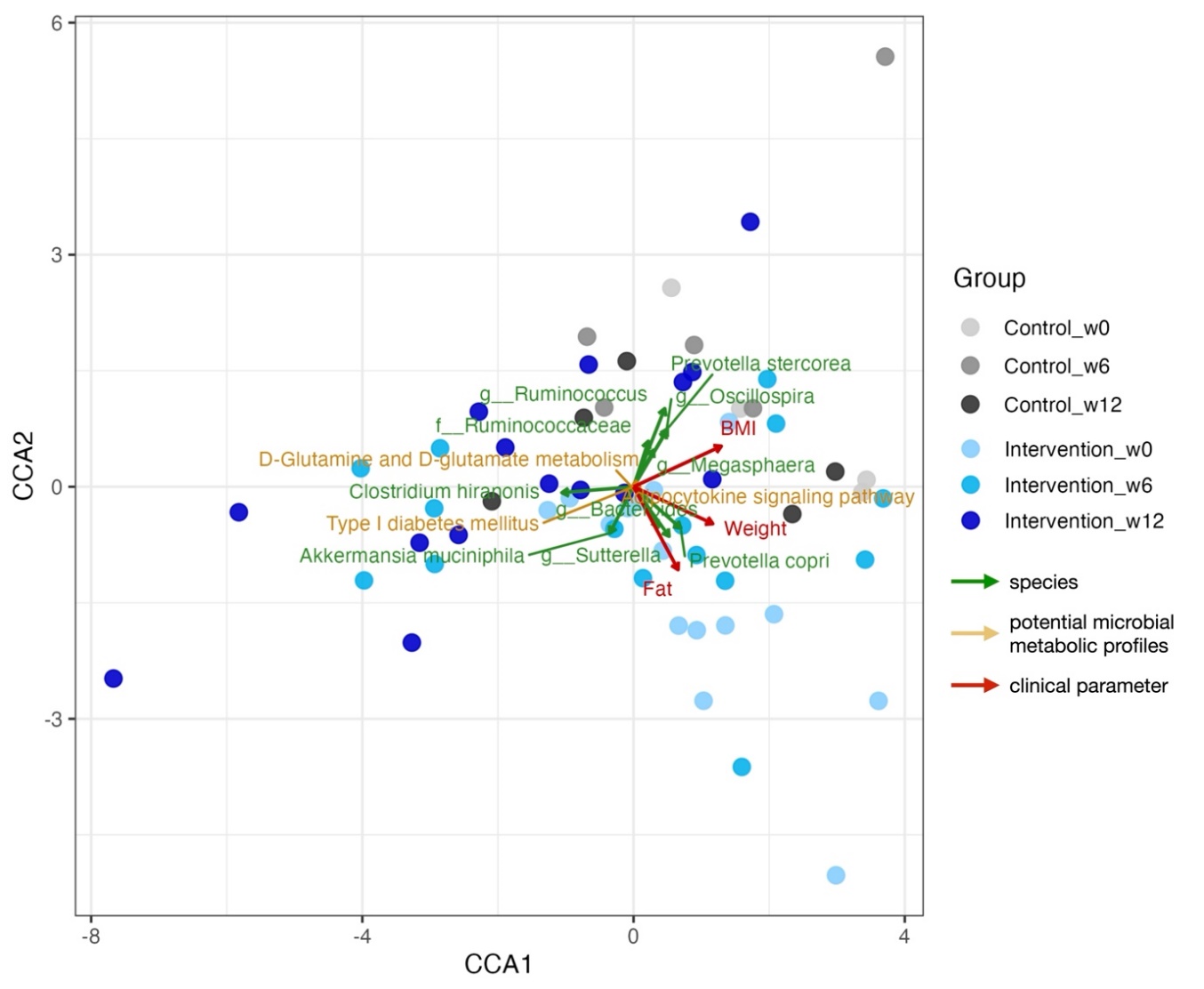
**

**Fig. S3.** Canonical correlation analysis (CCA) to reveal systemic relationships that link top 10 microbial species taxa with statistically significant microbial metabolic potentials and clinical parameters.

**Table S1.** AMOVA statistics of gut microbiota compositions at species OTU levels comparing between clinical groups (* represents *P* < 0.05).

| Comparing groups | | *P*-value |
| --- | --- | --- |
| Diabetes remission (P10, P12) | Control | 0.041* |
|  | Intervention | 0.072 |
| Could stop diabetes medicine (P4, P6, P10, P12) | Control | 0.088 |
|  | Intervention | 0.166 |

**Table S2.** Patient IDs, MiSeq sample codes, and Good’s coverage of microbiota at genus level.

| **Patient IDs** | **Group** | **Miseq codes** | **Coverage (%)** |
| --- | --- | --- | --- |
| P1_week0 | control | C01_W0 | 99.94 |
| P3_week0 | control | C02_W0 | 99.92 |
| P7_week0 | control | C03_W0 | 99.91 |
| P11_week0 | control | C04_W0 | 99.94 |
| P1_week6 | control | C01_W6 | 99.96 |
| P3_week6 | control | C02_W6 | 99.96 |
| P7_week6 | control | C03_W6 | 99.95 |
| P11_week6 | control | C04_W6 | 99.96 |
| P21_week6 | control | C05_W6 | 99.93 |
| P1_week12 | control | C01_W12 | 99.95 |
| P3_week12 | control | C02_W12 | 99.94 |
| P7_week12 | control | C03_W12 | 99.94 |
| P11_week12 | control | C04_W12 | 99.94 |
| P21_week12 | control | C05_W12 | 99.93 |
| P2_week0 | intervention | I01_W0 | 99.94 |
| P4_week0 | intervention | I02_W0 | 99.94 |
| P6_week0 | intervention | I03_W0 | 99.95 |
| P7_week0 | intervention | I04_W0 | 99.95 |
| P10_week0 | intervention | I05_W0 | 99.94 |
| P12_week0 | intervention | I06_W0 | 99.93 |
| P13_week0 | intervention | I07_W0 | 99.93 |
| P14_week0 | intervention | I08_W0 | 99.92 |
| P15_week0 | intervention | I09_W0 | 99.94 |
| P17_week0 | intervention | I10_W0 | 99.97 |
| P18_week0 | intervention | I11_W0 | 99.91 |
| P19_week0 | intervention | I12_W0 | 99.96 |
| P20_week0 | intervention | I13_W0 | 99.96 |
| P22_week0 | intervention | I14_W0 | 99.95 |
| P23_week0 | intervention | I15_W0 | 99.93 |
| P2_week6 | intervention | I01_W6 | 99.95 |
| P4_week6 | intervention | I02_W6 | 99.93 |
| P6_week6 | intervention | I03_W6 | 99.94 |
| P7_week6 | intervention | I04_W6 | 99.93 |
| P10_week6 | intervention | I05_W6 | 99.91 |
| P12_week6 | intervention | I06_W6 | 99.94 |
| P13_week6 | intervention | I07_W6 | 99.95 |
| P14_week6 | intervention | I08_W6 | 99.96 |
| P15_week6 | intervention | I09_W6 | 99.94 |
| P17_week6 | intervention | I10_W6 | 99.94 |
| P18_week6 | intervention | I11_W6 | 99.93 |
| P19_week6 | intervention | I12_W6 | 99.95 |
| P20_week6 | intervention | I13_W6 | 99.95 |
| P22_week6 | intervention | I14_W6 | 99.95 |
| P23_week6 | intervention | I15_W6 | 99.93 |
| P2_week12 | intervention | I01_W12 | 99.95 |
| P4_week12 | intervention | I02_W12 | 99.96 |
| P6_week12 | intervention | I03_W12 | 99.95 |
| P7_week12 | intervention | I04_W12 | 99.94 |
| P10_week12 | intervention | I05_W12 | 99.92 |
| P12_week12 | intervention | I06_W12 | 99.95 |
| P13_week12 | intervention | I07_W12 | 99.95 |
| P14_week12 | intervention | I08_W12 | 99.95 |
| P15_week12 | intervention | I09_W12 | 99.91 |
| P17_week12 | intervention | I10_W12 | 99.95 |
| P18_week12 | intervention | I11_W12 | 99.93 |
| P19_week12 | intervention | I12_W12 | 99.95 |
| P20_week12 | intervention | I13_W12 | 99.94 |
| P22_week12 | intervention | I14_W12 | 99.93 |
| P23_week12 | intervention | I15_W12 | 99.94 |
